# Automatic quantification of microvessel density in urinary bladder carcinoma

**DOI:** 10.1038/sj.bjc.6693399

**Published:** 1999-12

**Authors:** K Wester, P Ranefall, E Bengtsson, C Busch, P-U Malmström

**Affiliations:** Departments of Genetics & Pathology, Centre for Image Analysis, Urology, Uppsala University, Uppsala 75185, Sweden; Department of Pathology, University Hospital, Tromsö 9038, Norway

**Keywords:** microvessel density, angiogenesis, urinary bladder, carcinoma, image analysis, automatic quantification

## Abstract

Seventy-three TUR-T biopsies from bladder carcinoma were evaluated regarding microvessel density, defined as microvessel number (nMVD) and cross-section endothelial cell area (aMVD). A semi-automatic and a newly developed, automatic image analysis technique were applied in immunostainings, performed according to an optimized staining protocol. In 12 cases a comparison of biopsy material and the corresponding cystectomy specimen were tested, showing a good correlation in 11 of 12 cases (92%). The techniques proved reproducible for both nMVD and aMVD quantifications related to total tumour area. However, the automatic method was dependent on high immunostaining quality. Simultaneous, semi-automatic quantification of microvessels, stroma and epithelial fraction resulted in a decreased reproducibility. Quantification in ten images, selected in a descending order of MVD by subjective visual judgement, showed a poor observer capacity to estimate and rank MVD. Based on our results we propose quantification of MVD related to one tissue compartment. When staining quality is of high standard, automatic quantification is applicable, which facilitates quantification of multiple areas and thus, should minimize selection variability. © 1999 Cancer Research Campaign


*British Journal of Cancer* (1999) **81**(8), 1363–1370
© 1999 Cancer Research Campaign
Article no. bjoc.1999.0853

### Automatic quantification of microvessel density in urinary bladder carcinoma

**K Wester[1], P Ranefall[2], E Bengtsson[2], C Busch[4] and P-U Malmström[3]**

Departments of [1]Genetics & Pathology, [2]Centre for Image Analysis, [3]Urology, Uppsala University, 75185 Uppsala, Sweden; [4]Department of Pathology, University Hospital, 9038 Tromsö, Norway

**Summary** Seventy-three TUR-T biopsies from bladder carcinoma were evaluated regarding microvessel density, defined as microvessel number (nMVD) and cross-section endothelial cell area (aMVD). A semi-automatic and a newly developed, automatic image analysis technique were applied in immunostainings, performed according to an optimized staining protocol. In 12 cases a comparison of biopsy material and the corresponding cystectomy specimen were tested, showing a good correlation in 11 of 12 cases (92%). The techniques proved reproducible for both nMVD and aMVD quantifications related to total tumour area. However, the automatic method was dependent on high immunostaining quality. Simultaneous, semi-automatic quantification of microvessels, stroma and epithelial fraction resulted in a decreased reproducibility. Quantification in ten images, selected in a descending order of MVD by subjective visual judgement, showed a poor observer capacity to estimate and rank MVD. Based on our results we propose quantification of MVD related to one tissue compartment. When staining quality is of high standard, automatic quantification is applicable, which facilitates quantification of multiple areas and thus, should minimize selection variability.

© 1999 Cancer Research Campaign

**Keywords:** microvessel density; angiogenesis; urinary bladder; carcinoma; image analysis; automatic quantification

Angiogenesis or neovascularization is by definition formation of new capillaries from pre-existing blood vessels. The process of neovascularization in tumours is regulated by the activity of tumour cells, stromal cells and inflammatory cells (Folkman, 1995; Fox, 1997). There is evidence from in vivo and in vitro studies that tumour growth is dependent on neovascularization. This applies to both benign and malignant tumours and becomes critical after the tumour has grown beyond about 2 mm$^3$ (Folkman, 1990). Malignant and premalignant transitional epithelium, as well as cystitis cystica, have been shown to stimulate capillary proliferation on rabbit iris. In contrast, normal urothelium rarely had this effect (Chodak et al, 1980).

Microvessel density (MVD), as quantified in immunohistochemistry (IHC)-stained tissue sections, has proven to add prognostic information in a variety of tumours (Weidner, 1995*a*, 1995*b*; Vermeulen et al, 1996; Fox, 1997), including bladder carcinoma (Dickinson et al, 1994; Bochner et al, 1995; Jaeger et al, 1995; Hawke et al, 1996; Philp et al, 1996). However, contradictory results are also reported (Weidner, 1995*a*, 1995*b*; Vermeulen et al, 1996; Fox, 1997; Dinney et al, 1998). Although the counting technique described by Weidner et al (1992) is sometimes referred to as the ‘golden standard’, there is no consensus on how to perform MVD quantifications. Several approaches have been tried, which might, partly, explain the diverging results. Five main issues have to be resolved in a quantification strategy:

1. The choice of antibody and staining protocol: several antibodies have been tried, including factor VIII-related antigen, CD31, CD34, vimentin, *Ulex europaeus* agglutinin I, collagen IV, cathepsin B and histological stainings, such as haematoxylin-eosin and Masson’s trichrome. Studies performed to evaluate which antibody is best suited for use in microvessel quantifications have resulted in contradictory conclusions. Besides the choice of antibody, there are also differences between the staining protocols used, such as pretreatment methods and chromogens. Regarding quantification strategies, several approaches have been taken.
2. Definition of MVD: number of vessels, stained endothelial cell area, total vessel area including lumen or perimeter.
3. Choice of area for MVD quantification: the entire tumour area, hot spot, random, in areas of tumour invasion or in areas representative of the overall tumour grade.
4. Total magnification: ×40, ×50, ×100, ×160, ×200, ×400 and ×500 (The number of microscopic fields and corresponding size of the tumour area analysed vary greatly).
5. Quantification techniques: manual counting in the microscope and manual counting in the microscope using an ocular raster/grid, using Chalkley point eyepiece graticule, using a projection microscope with a grid square on the table, stereological quantification, image analysis equipment or by subjective grading of the vessel density. These issues have recently been reviewed by Vermeulen et al (1996) and others (Barbareschi et al, 1995*a*; Weidner, 1995*a*, 1995*b*; Fox, 1997)

In the present study, a new, automatic quantification method was used which automatically classifies an image into vessels and background, without observer interactivity. The technique has been recently described (Ranefall et al, 1999), proven stable regarding variations in light and focus settings, and showed a high concordance with manual counting of microvessels. For comparison, a semi-automatic quantification method was also used. This

*Received 11 December 1998*
*Revised 10 March 1999*
*Accepted 20 April 1999*

*Correspondence to:* K Wester method, in which reference points are subjectively chosen by the observer, has also been described previously (Ranefall et al, 1997), although not tested for microvessel quantification.

The aims of the present study were:

1. to develop an optimized IHC staining protocol suitable for automatic quantification of microvessels
2. to compare MVD in cystectomy specimens and preceding biopsies to assess the representativity of biopsy material
3. to evaluate both semi-automatic and automatic quantifications of MVD with respect to intra-observer reproducibility
4. to evaluate the observer capability in discriminating different MVD contents in neighbouring images.

### MATERIALS AND METHODS

### Patients

Two separate materials were analysed:

1. Twelve cases of bladder carcinoma, where both biopsy (transurethral resection material) and the corresponding cystectomy specimens were available, were obtained from the Department of Pathology, University Hospital Uppsala and used to test sampling representativity. All cases were cystectomized without preoperative treatment.
2. Biopsy specimens from 73 patients with invasive bladder carcinoma, stage T1–T4a (Malmstrom et al, 1996) were collected. The patients were randomized, regarding preoperative treatment, into a multicentre study (Nordic Cystectomy Study I) and were all subsequently cystectomized. The patients were recruited from ten hospitals in Sweden and the material was formalin-fixed and paraffin-embedded, according to standard procedures, at the respective local pathology department. The study was approved by ethical committee.

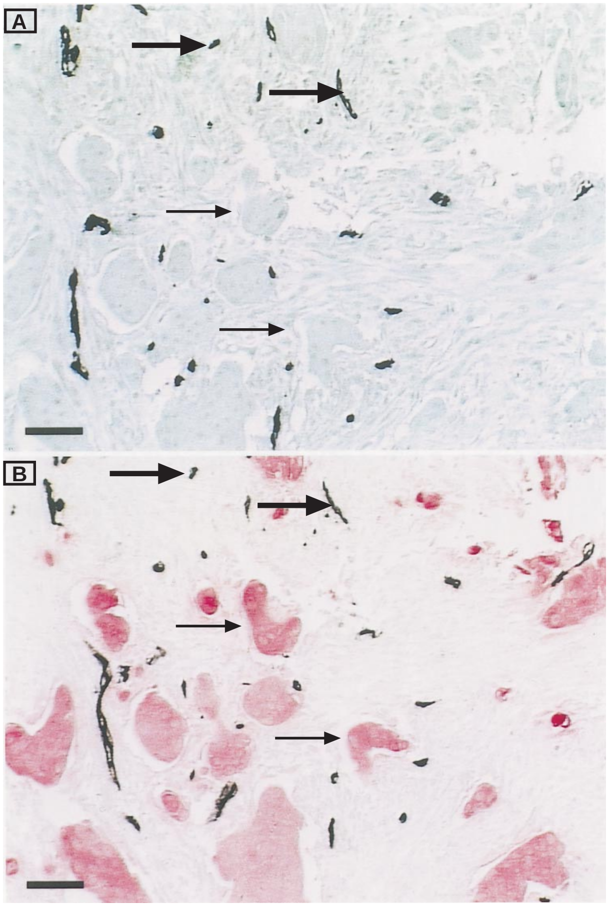

**Figure 1.** Microvessels highlighted by single IHC protocol (**A**) and by simultaneous IHC staining of epithelial cells, multiple IHC protocol (**B**). Fat arrows indicate microvessels and thin arrows tumour cell nests. Scale bars 50 µm.

### Immunohistochemistry

Parallel sets of slides were IHC stained for endothelial cells (single IHC) and for epithelial cells, as well as endothelial cells in the same section (multiple IHC) (Figure 1).

#### *Single IHC*

Paraffin sections were cut at 4-µm thickness and placed onto Super frost/plus® slides (Mentzel, Germany). Two mouse monoclonal antibodies, CD31 (clone JC70, Dako, Glostrup, Denmark) and CD34 (clone QBEND 10, Oxoid, Basingstoke, England) were used as a mixture, diluted 1:80 and 1:100 respectively and incubated for 1 h. Prior to IHC, heat mediated antigen retrieval (AR) was performed by boiling the slides in 0.01 M citrate buffer, pH 6.0, for 16 min at 750 W (Malmstrom et al, 1992) in a microwave oven (Whirlpool VIP34, Sweden). Both blocking for endogenous peroxidase in 0.3% hydrogen peroxide and preincubation in 10% normal rabbit serum (Dako), were diluted in phosphate-buffered saline (PBS) and incubated for 20 min. As link antibody, a biotinylated rabbit anti-mouse (Dako) was applied, followed by a peroxidase-labelled streptavidin–biotin complex (Dako), both diluted 1:200 and incubated for 30 min. The slides were developed in nickel-sulphate-enhanced DAB (Merck, Darmstadt, Germany; Sigma, St Louis, MO, USA respectively) for 6 min (Green et al, 1989) and counterstained in lightgreen (Merck). Finally, the slides were dehydrated through graded alcohols to xylene and mounted in organic mounting medium. Unless otherwise stated, reagents were diluted in 0.5% BSA-C (Aurion, Wageningen, The Netherlands) in PBS and incubations were performed at room temperature. Washings, for 3 × 10 min, between incubation steps were done in 0.05 M Tris, pH 7.6, containing 0.3 M sodium chloride and 0.1% Tween-20®.

#### *Double IHC*

The labelling of vessels was performed as above, except for the AR treatment, where 1 mM EDTA (Morgan et al, 1994), pH 8.0, was used instead of citrate buffer. After developing the first antibody (CD31/CD34) the slides were rinsed in tap water for 10 min before incubation in 1% normal mouse serum (Dako) in PBS for 20 min. To visualize the epithelial structures, an anti-cytokeratin antibody (clone AE1/AE3, Boehringer Mannheim, Mannheim, Germany) was applied for 16 h at 4°C. The link antibody and the streptavidin complex were diluted and incubated as above, except that alkaline phosphatase was used as labelling enzyme. Slides were developed in Vector red® (Vector, Burlingame, CA, USA) for 25 min, rinsed, counterstained in light green and mounted as above.

*© 1999 Cancer Research Campaign*

**Table 1** Description of antibodies against endothelial cells and cytokeratins evaluated in the study

| Antibody/clone | Optimal dilution | Company |
|---|---|---|
| Factor VIII/F8/86 | 1/50 | DAKO, Glostrup, Denmark |
| CD31/JC70 | 1/40 | DAKO, Glostrup, Denmark |
| CD34/QBEND10 | 1/50 | Oxoid/Unipath Ltd, Basingstoke, UK |
| FVIII+CD31+CD34 (mixture) | 1/100,1/80 & 1/100 | |
| CD31+CD34 (mixture) | 1/80 & 1/100 | |
| Cytokeratin 10, 17 & 18/MNF116 | 1/200 | DAKO, Glostrup, Denmark |
| Cytokeratin 8 | 1/100 | AB ImmunoDevelopLab, Sollentuna, Sweden |
| Cytokeratin 1–8, 10, 14–16 & 19/AE1/AE3 | 1/1000 | Boehringer & Mannheim, Mannheim, Germany |
| Cytokeratin 8 & 18/CAM | 1/50 | Beckton & Dickinson, Mountain View, CA, USA |
| Cytokeratin 18 | 1/100 | Sigma, St Louis, MO, USA |
| Cytokeratin 19 | 1/100 | DAKO, Glostrup, Denmark |
| Cytokeratin 17 | 1/20 | DAKO, Glostrup, Denmark |

**Table 2** Pretreatments tested in the study

| Pretreatment | Concentration | Temperature | Duration |
|---|---|---|---|
| Trypsinization | 0.1% | Room temperature | 30 min |
| Protease type VIII | 0.01% | Room temperature | 10 min |
| MW-citrate buffer[a] | 0.01 M | Boiling | $2 \times 7$ min |
| MW-EDTA[a] | 0.001 M | Boling | $2 \times 7$ min |

[a]Microwave irradiation at 750 W.

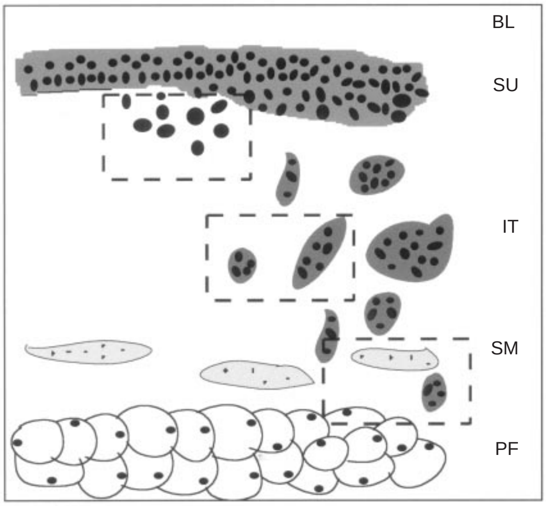

**Figure 2.** Schematic drawing of a tumour infiltrating bladder wall. Three tumour front windows typical for various stages of a tumour are shown. BL=bladder lumen, SU=superficial urothelium, IT=invasive tumour, SM=smooth muscle cell bundles and PF=perivesical fat cells.

### Image selection

In all 73 cases an area of 3–4 mm$^2$ was outlined. The criterion used for selecting these areas was the area subjectively judged as containing the highest number of microvessels at the deepest level of the bladder wall engaged by tumour, i.e. the tumour invasion front (Figure 2). The selection was done by three observers (KW, CB and PUM) by parallel examination of both Weigert–van Gieson and microvessel-stained slides. The selection process was performed before the quantifications started and without knowledge of patient outcome. Semi-automatic quantifications were also performed once without the requirement of tumour front localization, but in invasive tumour. Quantifications were always performed in 2 microscopic fields, except for one occasion where 10 microscopic fields were quantified, subjectively ranged in a descending order regarding MVD. The size of the recorded images corresponded to 0.22 mm$^2$ per image.

### Image acquisition

The $756 \times 572$ pixel colour images with $3 \times 256$ grey levels were grabbed by a Sony DXC-151 colour video camera attached to a standard Olympus BH-10 microscope, using 20× objective. This gave a final magnification of ×50 and a pixel size of about 0.8 μm for a wavelength of 550 nm. For all images, Köhler illumination was maintained and the aperture iris diaphragm ring was fixed to 0.5.

### Classification of video images and definition of variables

The semi-automatic counting of vessel number was an algorithm allowing the operator to label each structure, subjectively judged as a microvessel, in an image and the result was subsequently presented as the total number of labelled objects per image. Any stained isolated endothelial cell or cohesive endothelial cell cluster was considered to represent a single countable microvessel. Vessel

© 1999 Cancer Research Campaign

**Table 3** Chromogens tested in the study

| Chromogen | Company | Colour |
|---|---|---|
| DAB | Sigma, St Louis, MO, USA | Brown-red |
| Nickel-enhanced DAB | Sigma, St Louis, MO, USA | Black |
| AEC | Sigma, St Louis, MO, USA | Red-brown |
| VIP® | Vector, Burlingame CA, USA | Purple |
| Fast red® | Vector, Burlingame CA, USA | Red |
| Fast blue® | Vector, Burlingame CA, USA | Blue |
| SG® | Vector, Burlingame CA, USA | Grey-black |
| New fuchsin® | DAKO, Glostrup, Denmark | Red |

lumen or red blood cells were not necessary for a structure to be defined as a microvessel. All muscular arteries were excluded.

The semi-automatic vessel area quantifications were based on manually selected reference points, representing the respective colour subclass in an image. Microvessel area, aMVD, was defined as stained endothelial cell surface area, excluding vessel lumen.

The automatic quantification was performed without influence from the operator. No interactive steps were involved besides the selection of respective image. In contrast to the semi-automatic method, the area quantification here included information describing both stained endothelial cell area and total vessel area. From here on, stained endothelial cell area is refered to as aMVD.

The software was developed at the Centre for Image Analysis, Uppsala University, Sweden and is presented in detail elsewhere (Ranefall et al, 1997, 1998).

### RESULTS

### Material 1

#### *IHC protocol evaluation*

Four different antibodies to endothelial cells; and seven to cytokeratin were tested. Staining of endothelial cells were also tested using cocktails, composed of two or three antibodies each, in varying combinations and concentrations. Different pretreatment protocols, chromogens and counterstains were also tested. Details are shown in Tables 1–3. All stainings were evaluated by two observers, regarding specificity, sensitivity, intensity, resolution and contrast in the respective staining alternative. One single IHC protocol (1 × IHC) was chosen because of its excellent outlining of vessels, with a sharp contrast towards the surrounding tissue. A mixture of CD31 and CD34 was judged as the best alternative for visualizing the vessels. The double IHC protocol (2 × IHC) chosen showed an almost equally high contrast and was also the superior alternative for the visualization of the epithelial compartment. The latter was mainly an effect of the antigen retrieval in EDTA-buffer which further enhanced the cytokeratin immunostaining. The vessel staining (CD31/CD34 mixture) was only marginally effected by the exchange of citrate-buffer for EDTA. The two protocols are described in detail in Materials and Methods and the staining outcome illustrated in Figure 1.

#### *Area selection in biopsies compared to cystectomy specimens*

In this study it is stated that MVD is quantified in microvessel 'hot spots' in the tumour front (Figure 2). The question is, however: is it possible, in fragmented biopsy specimens, to select a well-defined area that corresponds to the 'reality' in cystectomy specimens? Therefore, nMVD and aMVD were quantified in parallel biopsy and cystectomy specimens. As visualized in Figure 3A an excellent agreement for nMVD was found in 11 of 12 cases (92%), whereas aMVD showed a good concordance in eight of 12 cases (67%) (Figure 3B).

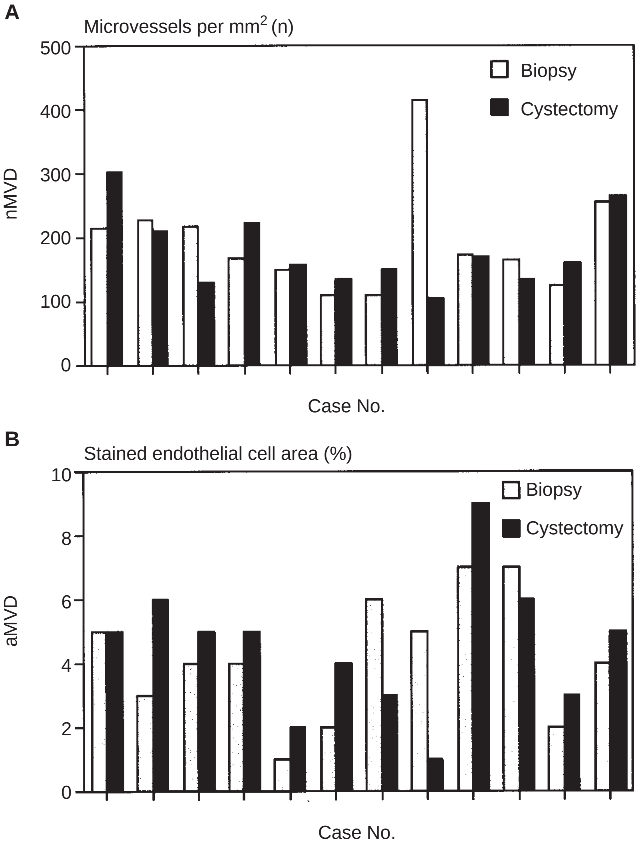

**Figure 3**. (**A**) Comparison of nMVD per total tumour area in parallel biopsy and cystectomy specimens using semi-automatic quantification. (**B**) Comparison of aMVD per total tumour area in parallel TUR and cystectomy specimens using semi-automatic quantification.

### Material 2

#### *Evaluation of automatic and semi-automatic quantification*

*Semi-automatic quantification* of nMVD was highly reproducible ($r$ = 0.88) when related to total tumour area and applied on

© 1999 Cancer Research Campaign

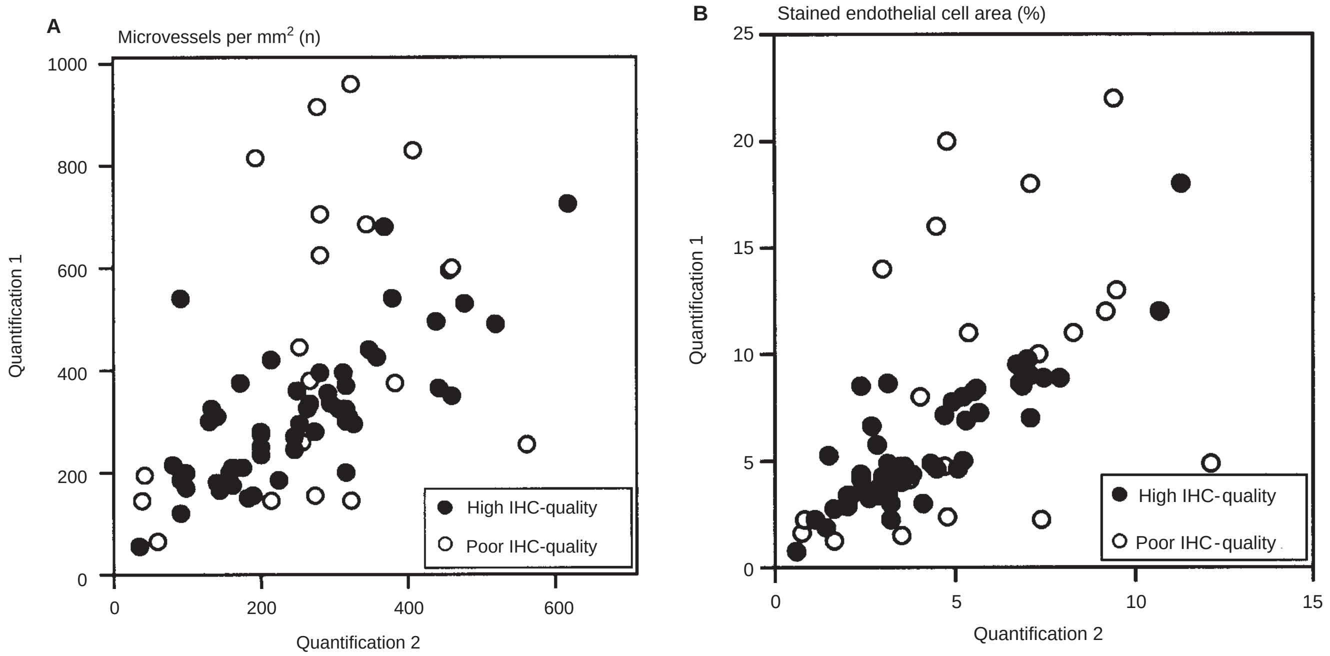

**Figure 4.** (**A**) Automatic quantification of nMVD per total tumour area performed at two occasions, illustrating the influence of poor IHC staining quality on reproducibility. (**B**) Automatic quantification of aMVD per total tumour area performed at two occasions, illustrating the influence of poor IHC staining quality on reproducibility.

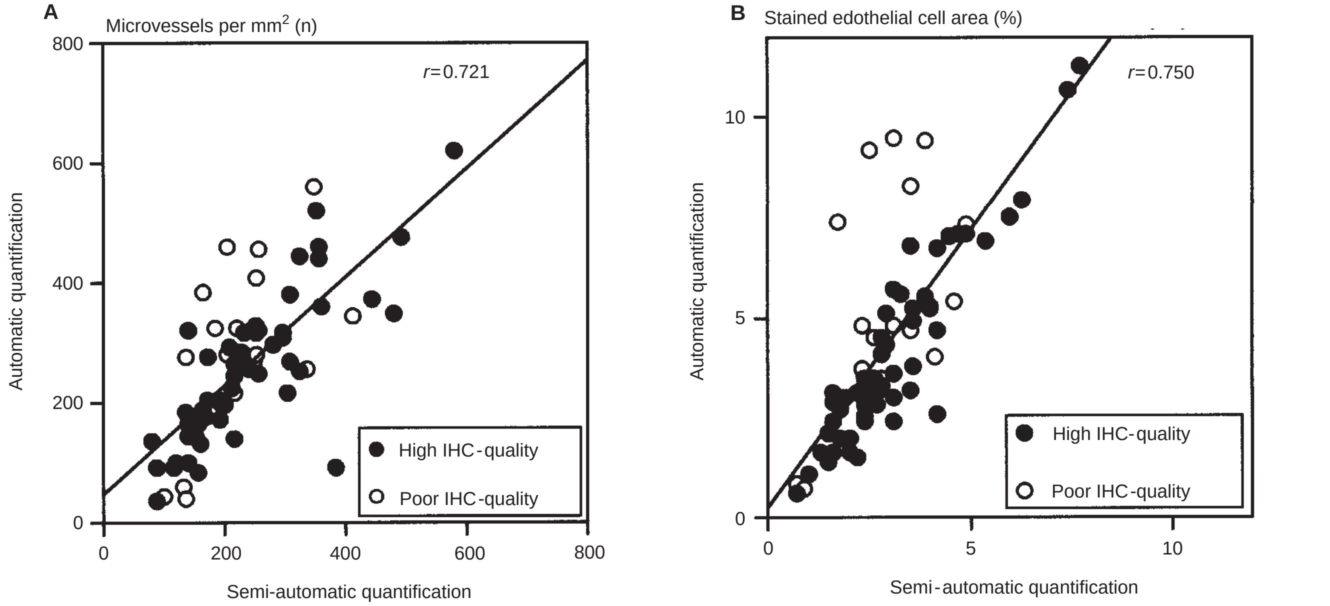

**Figure 5.** (**A**) Association between semi-automatic and automatic quantification of nMVD, per total tumour area, including all 73 cases. (**B**) Association between semi-automatic and automatic quantification of aMVD, per total tumour area, including all 73 cases.

1 × IHC stainings. The corresponding number for aMVD was 0.72. When these quantifications were performed on 2 × IHC stainings, a decreased reproducibility was observed for aMVD ($r$ = 0.56) whereas nMVD reproducibility was only marginally affected ($r$ = 0.85). In this analysis, new images were recorded for each quantification round. If exactly the same images were quantified on two occasions, the reproducibility was increased for nMVD and aMVD ($r$ = 0.97 and 0.88).

Simultaneous quantification of three compartments, vessels, epithelium/tumour and stroma, outlined by the double IHC-protocol and expressing MVD related to stromal fraction, was less reproducible, $r$ = 0.51 for nMVD and 0.43 for aMVD.

*The automatic quantifications* of nMVD and aMVD, applied to single IHC stainings, showed a lower reproducibility compared to the semi-automatic quantification, $r$ = 0.53 and 0.65 respectively. However, when 19 cases judged as of poor IHC staining quality were excluded, the corresponding figures were 0.73 and 0.87 (Figure 4 A, B). The criteria for exclusion were based on a subjective judgement of the same staining qualities as described in the 'IHC protocol evaluation' section. This was performed by one

*© 1999 Cancer Research Campaign*

observer on two occasions resulting in a concordance in 71 of 73 cases.

There was a good agreement between semi-automatic and automatic quantifications for both nMVD and aMVD respectively, $r$ = 0.72 and 0.75 (Figure 5 A, B). If the 19 cases of poor IHC staining quality were excluded, the correlation improved ($r$ = 0.80 for nMVD and 0.92 for aMVD).

It was not possible to perform three-compartment quantification automatically.

### *Assessment of the intra-observer reliability regarding selection of the highest MVD areas*

Based on the subjective impression of MVD, ten images from each case were selected, ranged in a descending order (1 to 10). Subsequently, the subjective ranking of the images were compared to the results from the corresponding semi-automatic quantification. Regarding nMVD in 20 of 73 cases (27%), the subjective impression was in agreement with the semi-automatic quantification. The corresponding fraction for aMVD were 16 of 73 (22%). In the discrepant cases, one or both of the images with the highest MVD, as quantified semi-automatically, was found in images ranked from 3 to 10 by subjective impression. The correlation between results from quantification of 2 images (subjectively ranked as 1 and 2) and the total 10 was good, $r$ = 0.67 and 0.77 for nMVD and aMVD respectively.

Quantifications in hotspot areas, chosen without further specification within invasive tumour, was performed once. The results were poorly correlated to, and generally higher than, quantifications performed in tumour front areas (results not shown). Hotspot quantifications were performed by semi-automatic techniques and no attempts were made to evaluate the reproducibility.

### DISCUSSION

We have previously applied computer-based image analysis technique for malignancy grading of urinary bladder carcinoma with promising results regarding reproducibility (Jarkrans et al, 1995; Choi et al, 1997). In this study, we used a similar methodological approach to assess MVD.

The validity of quantitative results from IHC-stainings are sometimes questioned (Wold et al, 1989; Wagner, 1993; Lambkin et al, 1994). Recently, several investigators have compared sensitivity and specificity for different antibodies against endothelial cells (Hollingsworth et al, 1995; Arakawa et al, 1997; Lee et al, 1997; Martin et al, 1997; Duarte et al, 1998), but additional factors such as pretreatment, choice of chromogen and counterstaining are seldom evaluated. Based on an extensive screening of available antibodies, pretreatments and chromogens, optimized IHC-staining protocols for visualization of the histological compartments of interest were developed. Despite our efforts, using the automatic quantification technique, 19 cases had to be excluded in this study. Using CD31 or CD34 as single antibody, instead of a mixture, did not alter the staining result. It is therefore reasonable to assume that fixation and/or histoprocessing is responsible for the poor immunostaining results. Of the excluded cases, 13 originated from three of the ten hospitals included in the study. The exclusion rate for specimens from these three hospitals were 42, 60 and 72% respectively. This illustrates clearly one of the major problems in standardization of IHC. The rate of poor quality stainings probably increased because of the fact that the IHC-staining protocols were developed and optimized on material from one of the contributing hospitals and subsequently applied on material from nine other hospitals. Potential variations in fixation time, histoprocessing regimens and material storing conditions between laboratories are known to effect the IHC-staining quality (Leong and Gilham, 1989; Fisher et al, 1994; McDermott et al, 1997). The necessity of an initial subjective judgment of the staining quality is a limiting factor in the development of an objective automatic quantification method.

We suggest that the prognostic implicit of tumour angiogenesis relates to the amount and mode of growth of the tumours. Therefore, to express MVD related to tumour stroma area or epithelial fraction of the tumour might add important prognostic information. To our knowledge no such attempts have been previously made. In this study an IHC-staining protocol was developed for the staining of both the microvessels alone and the tumour cells alone. These three-compartmental quantifications performed were associated with a decreased reproducibility. However, these stainings proved helpful in the screening for appropriate areas, here tumour front, for MVD quantification. The double IHC and single IHC stainings were equally suited for semi-automatic quantifications of nMVD related to total tumour area, whereas semi-automatic aMVD quantifications were less suited, regarding reproducibility, when applied to multiple IHC stainings.

Studies of MVD in urinary bladder carcinoma have investigated biopsy (Dickinson et al, 1994; Philp et al, 1996; Nakanishi et al, 1997) and cystectomy specimens (Bochner et al, 1995; Jaeger et al, 1995; Grossfeld et al, 1997). The latter should be more suitable as the relation between tumour and vasculature is more easily assessed, presumably in a more representative and reproducible way. This is further stressed by Bochner et al (1995) who state that most bladder tumours exhibit a large degree of heterogeneity with respect to microvessel density. However, the preceding transurethral resection biopsy is the basis for treatment decision and prognostication. Also only a minority of newly diagnosed cases are operated with cystectomy. Therefore, for prognostication in clinical practice any marker has to be evaluated in the biopsy specimen.

Comparing biopsy material and corresponding cystectomy specimen, a major discrepancy in MVD was detected in one of 12 cases. This case exhibited a low MVD in the cystectomy specimen (Figure 3 A, B). An explanation may be that the most vascularized tumour fraction was removed at biopsy. There are discrepancies between the cystectomy specimens and the fragmented biopsy specimens regarding evaluation of MVD. Cystectomy specimens provide a better representation of the tumour growth pattern. ‘Keyhole’ view in biopsy specimens illustrates the importance and problems of representativity. More extensive MVD quantification in biopsies might also be a way to minimize the effects of heterogeneity.

Different quantification strategies have been reported. In most studies different manual counting techniques have been proposed (Weidner et al, 1991; Dickinson et al, 1994; Jaeger et al, 1995) and sometimes also compared with semi-automatic, image-analysis-aided quantification of stained endothelial cell pixel area (Barbareschi et al, 1995*b*; Fox et al, 1995; Kohlberger et al, 1996). The reproducibility has been reported as moderate to high, at both intra- and inter-observer level (Bochner et al, 1995; de Jong et al, 1995; Penfold et al, 1996; Philp et al, 1996). All methods described hitherto are influenced by subjective decisions made by the observer. To avoid these potential sources of intra- and inter-observer variability, an automatic method was developed and evaluated in comparison to semi-automatic quantification

 © 1999 Cancer Research Campaign methods. The study was designed to analyse the effects of observer quantification interactivity. By minimizing the area selection variability, this could be done more explicitly. Thus, all quantifications were performed in a strictly defined histological area, previously demarcated on respective slides. In this way the intra-observer reproducibility was considered as an assessment of the quantification method stability. No attempts were made to evaluate inter-observer or area screening reproducibility.

When semi-automatic quantification of nMVD and aMVD is performed at two occasions on identical images the reproducibility is high. The reproducibility for the automatic quantification here may be regarded as perfect, even if variations in light settings and focus are considered, as we have previously shown (Ranefall et al, 1998). When quantifications were performed within an outlined area, but with a potential variance in the choice of image, they were slightly less reproducible for the semi-automatic method. For the automized quantifications this was more evident. This was due to the latter method's vulnerability to reduced staining quality and inability to recognize obvious artefacts. Exclusion of cases with visible poor IHC-staining quality resulted in a reproducibility similar to the semi-automatic quantifications.

As observed, the intra-operator variability is more pronounced regarding estimation of area fractions than object recognition and counting. This is also evident when automatic and semi-automatic quantification of aMVD are compared, where elimination of the intra-operator variability using an automized method improves the reproducibility (Figure 5B). On the contrary, the semi-automized method was superior in object recognition and counting, especially when specimens of poor IHC stainings were not excluded (Figure 5A).

The results from the semi-automatic quantifications indicate that the subjective selection of images, even within a small outlined area, influenced the intra-observer reproducibility. This was also found for the interactive quantification steps, i.e. counting and reference point selection.

In a study performed in breast carcinoma Martin et al (1997) concluded that finding the area with the highest MVD appeared to be the most subjective step in microvessel quantification methodology. This was based on results that showed an efficiency of only 20% in finding the 'hottest spot' in the first chosen microscopic field out of 10 counted. In this study a similar test resulted in a slightly higher efficiency (27 and 22% for nMVD and aMVD respectively). Still, in most cases an increased MVD was observed when multiple areas were used to identify the hot spot images. The correlation between MVD from 2 images only and the true highest 2 out of 10 was high regarding nMVD, but lower for aMVD. Considering all images were selected within a limited area, the consequence was that quantification of an extensive tumour fraction led to increased hot spot recognition. Even when an apparently homogeneous fraction within a tumour is quantified, observer subjectivity in the selection of images generates method variability. Using the automatic quantification technique described here, multiple areas can be rapidly evaluated. The automized technique reduces the quantification time up to three to four times compared to interactive measurements (data not shown) and simultaneously provides both number and area estimates of the microvessels.

The medical application of computerized image analysis is a progressing field. An increased awareness of the need for objective quantification methods together with improved equipment performance will further promote this progress. The expensive equipment has earlier restricted the accessibility to computerized image analysis techniques to major hospitals and laboratories. Today, high performance systems are available for less than £10 000, thus increasing the number of potential users.

### CONCLUSIONS

Based on our results, on reproducibility, we propose the following schedule for assessment of MVD in urinary bladder carcinoma biopsies:

- To perform an initial subjective evaluation of the IHC staining quality, excluding specimens of poor quality.
- To evaluate MVD related to total tumour area using a single chromogen IHC staining protocol.
- To use an automatic quantification technique that simultaneously and rapidly provides information of both number and total vessel area as well as stained endothelial cell area.
- To quantitate in a large proportion of the tumour fraction of interest.

This method is now being tested regarding its prognostic value, specifically considering the choice of assessment region in the tumour. Strictly defined regions for assessment of MVD is, in addition, crucial to an improvement of inter-observer reproducibility.

### ACKNOWLEDGEMENT

This work was supported by Linnérs-Hagstrands fund and grant from the Swedish Cancer Society (grant 2323–B97–11XAA).

### REFERENCES

Arakawa A, Soh S, Chakraborty S, Scardino PT and Wheeler TM (1997). Prognostic significance of angiogenesis in clinically localized prostate cancer (staining for Factor VIII-related antigen and CD34 antigen). *Prost Cancer Prost Dis* **1**: 32–38

Barbareschi M, Gasparini G, Morelli L, Forti S and Dalla Palma P (1995*a*) Novel methods for the determination of the angiogenic activity of human tumors. *Breast Cancer Res Treat* **36**: 181–192

Barbareschi M, Weidner N, Gasparini G, Morelli L, Forti S, Eccher C, Fina P, Caffo O, Leonardi E, Mauri F, Bevilacqua P and Dalla Palma P (1995*b*) Microvessel density quantification in breast carcinomas. Assessment by light microscopy vs. a computer-aided image analysis system. *Appl Immunohistochem* **3**: 75–84

Bochner BH, Cote RJ, Weidner N, Groshen S, Chen SC, Skinner DG and Nichols PW (1995) Angiogenesis in bladder cancer: relationship between microvessel density and tumor prognosis. *J Natl Cancer Inst* **87**: 1603–1612

Chodak GW, Haudenschild C, Gittes RF and Folkman J (1980) Angiogenic activity as a marker of neoplastic and preneoplastic lesions of the human bladder. *Ann Surg* **192**: 762–771

Choi HK, Jarkrans T, Bengtsson E, Vasko J, Wester K, Malmstrom PU and Busch C (1997) Image analysis based grading of bladder carcinoma. Comparison of object, texture and graph based methods and their reproducibility. *Anal Cell Pathol* **15**: 1–18

de Jong JS, van Diest PJ and Baak JP (1995) Heterogeneity and reproducibility of microvessel counts in breast cancer. *Lab Invest* **73**: 922–926

Dickinson AJ, Fox SB, Persad RA, Hollyer J, Sibley GN and Harris AL (1994) Quantification of angiogenesis as an independent predictor of prognosis in invasive bladder carcinomas. *Br J Urol* **74**: 762–726

Dinney CP, Babkowski RC, Antelo M, Perrotte P, Liebert M, Zhang HZ, Palmer J, Veltri RW, Katz RL and Grossman HB (1998) Relationship among cystectomy, microvessel density and prognosis in stage T1 transitional cell carcinoma of the bladder. *J Urol* **160**: 1285–1290

Duarte IG, Bufkin BL, Pennington MF, Gal AA, Cohen C, Kosinski AS, Mansour KA and Miller JI (1998) Angiogenesis as a predictor of survival after surgical

© 1999 Cancer Research Campaign 

resection for stage I non-small-cell lung cancer. *J Thorac Cardiovasc Surg* **115**: 652–658
Fisher CJ, Gillett CE, Vojtesek B, Barnes DM and Millis RR (1994) Problems with p53 immunohistochemical staining: the effect of fixation and variation in the methods of evaluation. *Br J Cancer* **69**: 26–31
Folkman J (1990) What is the evidence that tumors are angiogenesis dependent? [editorial]. *J Natl Cancer Inst* **82**: 4–6
Folkman J (1995) Angiogenesis in cancer, vascular, rheumatoid and other disease. *Nat Med* **1**: 27–31
Fox SB (1997) Tumour angiogenesis and prognosis. *Histopathology* **30**: 294–301
Fox SB, Leek RD, Weekes MP, Whitehouse RM, Gatter KC and Harris AL (1995) Quantitation and prognostic value of breast cancer angiogenesis: comparison of microvessel density, Chalkley count, and computer image analysis. *J Pathol* **177**: 275–283
Green MA, Sviland L, Malcolm AJ and Pearson AD (1989) Improved method for immunoperoxidase detection of membrane antigens in frozen sections. *J Clin Pathol* **42**: 875–880
Grossfeld GD, Ginsberg DA, Stein JP, Bochner BH, Esrig D, Groshen S, Dunn M, Nichols PW, Taylor CR, Skinner DG and Cote RJ (1997) Thrombospondin-1 expression in bladder cancer: association with p53 alterations, tumor angiogenesis, and tumor progression. *J Natl Cancer Inst* **89**: 219–227
Hawke CK, Delahunt B and Davidson PJT (1996) The prognostic value of microvessel density in bladder cancer. *J Urol* **155**: 690A (abstract 1517)
Hollingsworth HC, Kohn EC, Steinberg SM, Rothenberg ML and Merino MJ (1995) Tumor angiogenesis in advanced stage ovarian carcinoma. *Am J Pathol* **147**: 33–41
Jaeger TM, Weidner N, Chew K, Moore DH, Kerschmann RL, Waldman FM and Carroll PR (1995) Tumor angiogenesis correlates with lymph node metastases in invasive bladder cancer. *J Urol* **154**: 69–71
Jarkrans T, Vasko J, Bengtsson E, Choi HK, Malmstrom PU, Wester K and Busch C (1995) Grading of transitional cell bladder carcinoma by image analysis of histological sections. *Anal Cell Pathol* **8**: 135–158
Kohlberger PD, Obermair A, Sliutz G, Heinzl H, Koelbl H, Breitenecker G, Gitsch G and Kainz C (1996) Quantitative immunohistochemistry of factor VIII-related antigen in breast carcinoma: a comparison of computer-assisted image analysis with established counting methods. *Am J Clin Pathol* **105**: 705–710
Lambkin HA, Mothersill CM and Kelehan P (1994) Variations in immunohistochemical detection of p53 protein overexpression in cervical carcinomas with different antibodies and methods of detection. *J Pathol* **172**: 13–18
Lee AHS, Happerfield LC and Bobrov LG (1997) Comparison of four endothelial markers for assessing angiogenesis in carcinoma of the breast. *J Cell Pathol* **2**: 67–73
Leong AS-Y and Gilham PN (1989) The effects of progressive formaldehyde fixation on preservation of tissue antigens. *Pathology* **21**: 266–268
McDermott N, Farah N, Milburn C, Butler D, Kay E, Barry Walsh C and Leader M (1997) MIB1 staining in archival material: problems with immunostaining in older paraffin embedded tissue may limit its predictive value. *J Cell Pathol* **2**: 113–115
Malmstrom PU, Wester K, Vasko J and Busch C (1992) Expression of proliferative cell nuclear antigen (PCNA) in urinary bladder carcinoma. Evaluation of antigen retrieval methods. *Apmis* **100**: 988–992
Malmstrom PU, Rintala E, Wahlqvist R, Hellstrom P, Hellsten S and Hannisdal E (1996) Five-year followup of a prospective trial of radical cystectomy and neoadjuvant chemotherapy: Nordic Cystectomy Trial I. The Nordic Cooperative Bladder Cancer Study Group [see comments]. *J Urol* **155**: 1903–1906
Martin L, Green B, Renshaw C, Lowe D, Rudland P, Leinster SJ and Winstanley J (1997) Examining the technique of angiogenesis assessment in invasive breast cancer. *Br J Cancer* **76**: 1046–1054
Morgan JM, Navabi H, Schmid KW and Jasani B (1994) Possible role of tissue-bound calcium ions in citrate-mediated high-temperature antigen retrieval. *J Pathol* **174**: 301–307
Nakanishi K, Hiroi S, Kawai T and Torikata C (1997) Expression of platelet-derived growth-factor B-chain mRNA and tumor angiogenesis in invasive transitional cell carcinoma of the upper urinary tract. *Mod Pathol* **10**: 341–347
Penfold CN, Partridge M, Rojas R and Langdon JD (1996) The role of angiogenesis in the spread of oral squamous cell carcinoma. *Br J Oral Maxillofac Surg* **34**: 37–41
Philp EA, Stephenson TJ and Reed MW (1996) Prognostic significance of angiogenesis in transitional cell carcinoma of the human urinary bladder. *Br J Urol* **77**: 352–357
Ranefall P, Egevad L, Nordin B and Bengtsson E (1997) A new method for segmentation of colour images applied to immunohistochemically stained cell nuclei. *Anal Cell Pathol* **15**: 145–156
Ranefall P, Wester K, Busch C, Malmström P-U and Bengtsson E (1998) Automatic quantification of microvessels using unsupervised image analysis. *Anal Cell Pathol* **17**: 83–92
Vermeulen PB, Gasparini G, Fox SB, Toi M, Martin L, McCulloch P, Pezzella F, Viale G, Weidner N, Harris AL and Dirix LY (1996) Quantification of angiogenesis in solid human tumours: an international consensus on the methodology and criteria of evaluation. *Eur J Cancer* **32a**: 2474–2484
Wagner BM (1993) To immunostain or not to immunostain, that is the question [editorial]. *Mod Pathol* **6**: 113
Weidner N (1995*a*) Current pathologic methods for measuring intratumoral microvessel density within breast carcinoma and other solid tumors. *Breast Cancer Res Treat* **36**: 169–180
Weidner N (1995*b*) Intratumor microvessel density as a prognostic factor in cancer [comment]. *Am J Pathol* **147**: 9–19
Weidner N, Semple JP, Welch WR and Folkman J (1991) Tumor angiogenesis and metastasis – correlation in invasive breast carcinoma. *N Engl J Med* **324**: 1–8
Weidner N, Folkman J, Pozza F, Bevilacqua P, Allred EN, Moore DH, Meli S and Gasparini G (1992) Tumor angiogenesis: a new significant and independent indicator in early-stage breast carcinoma. *J Natl Cancer Inst* **84**: 1875–1887
Wold LE, Corwin DJ, Rickert RR, Pettigrew N and Tubbs RR (1989) Interlaboratory variability of immunohistochemical stains. Results of the cell markers survey of the College of American Pathologists. *Arch Pathol Lab Med* **113**: 680–683

 *© 1999 Cancer Research Campaign*
